# Delta-Band EEG Microstate Dynamics as Promising Candidate Markers of Central Vertigo Severity

**DOI:** 10.3390/brainsci16020143

**Published:** 2026-01-28

**Authors:** Jiedong Nan, Yanru Bai, Haoran Jiang, Yuncheng Zhao, Yang Xiao, Guangjian Ni

**Affiliations:** 1 Academy of Medical Engineering and Translational Medicine, Tianjin University, Tianjin 300072, China; jiedongnan@tju.edu.cn (J.N.); haoranjiang@tju.edu.cn (H.J.); 2022202315@tju.edu.cn (Y.Z.); xy_shawn@tju.edu.cn (Y.X.); niguangjian@tju.edu.cn (G.N.); 2 Haihe Laboratory of Brain-Computer Interaction and Human-Machine Integration, Tianjin 300392, China; 3 State Key Laboratory of Advanced Medical Materials and Devices, Tianjin 300072, China; 4 Tianjin Key Laboratory of Brain Science and Neuroengineering, Tianjin 300072, China

**Keywords:** central vertigo, EEG, microstates, delta band, disease severity

## Abstract

**Background/Objectives:** Central vertigo (CV) lacks objective biomarkers for severity assessment. This study examined whether resting-state EEG microstate dynamics across frequency bands can distinguish CV severity. **Methods:** Resting-state EEG was recorded from 50 patients with stroke-related CV and 31 healthy controls. Patients were classified as moderate (MD, *n* = 31) or severe (SV, *n* = 19) based on Dizziness Handicap Inventory scores. Microstate analysis was performed in the delta, theta, alpha, and beta bands to assess microstate topographies, temporal parameters, and transition probabilities. Correlations with clinical measures and receiver operating characteristic analyses were conducted. **Results:** CV patients showed severity-dependent alterations in EEG microstate dynamics, most pronounced in the delta band. Delta-band microstate transition probabilities correlated significantly with symptom severity and balance confidence. The delta-band transition from microstate C to microstate B accurately differentiated MD from SV patients (AUC = 0.983). **Conclusions:** Delta-band EEG microstate transition dynamics reflect network dysfunction in CV and may serve as promising candidate biomarkers for CV severity stratification.

## 1. Introduction

CV represents a diagnostically challenging subset of vestibular disorders arising from dysfunction within brainstem, cerebellar, or cortical vestibular pathways rather than peripheral end-organs. Although constituting approximately 10–20% of acute vestibular presentations in emergency settings, CV demonstrates greater diagnostic complexity compared with peripheral vestibular disorders due to overlapping symptoms, subtle neurological signs, and the lack of reliable objective biomarkers [1,2]. Importantly, CV frequently reflects serious underlying pathologies, such as posterior circulation stroke, demyelinating disease, or intracranial tumors, requiring urgent clinical intervention [3,4]. Current vestibular assessments primarily rely on behavioral tests and vestibular-evoked myogenic potentials, which mainly evaluate peripheral vestibular function and brainstem reflexes, while structural neuroimaging such as magnetic resonance imaging (MRI) remains indispensable for detecting gross lesions but offers limited functional insights into distributed cortical network dysfunction [5,6]. Functional neuroimaging studies have shown that vestibular processing engages a distributed temporo-parieto-insular network centered on the parieto-insular vestibular cortex (PIVC) with extensive integration across multisensory, attentional, and default-mode networks [4,7,8]. The vestibular cortex itself is characterized as a multisensory hub that integrates visual, proprioceptive, and vestibular cues to support spatial orientation and balance [9]. However, the limited temporal resolution of hemodynamic imaging modalities precludes characterization of the rapid neural dynamics underlying vestibular processing.

EEG offers a complementary approach capable of capturing neural activity with millisecond temporal precision, thereby enabling the analysis of rapid oscillatory dynamics across distributed brain networks [10]. Neural oscillations across distinct frequency bands play a central role in large-scale network coordination, with pathological increases in low-frequency activity—particularly in the delta band—widely recognized as markers of disrupted cortical function and impaired sensory gating [11,12,13,14]. Under normal waking conditions, delta oscillations are relatively suppressed, whereas their enhancement reflects cortical deafferentation, attentional dysfunction, and impaired inter-sensory integration—all of which are relevant to severe vestibular disturbance. Beyond conventional spectral analyses, EEG microstate analysis provides a powerful framework for examining the temporal dynamics of large-scale brain networks. Microstates represent brief periods of stable scalp topographies lasting approximately 60–120 ms that can be reliably categorized into canonical classes (A–D), each associated with distinct functional networks, including visual, salience/default-mode, and attentional systems [15,16]. Alterations in microstate temporal properties and transition probabilities have been reported across numerous neuropsychiatric and neurodegenerative disorders, reflecting disrupted network coordination [17]. However, microstate analysis has been scarcely applied to vestibular disorders, and systematic investigations of microstate dynamics in CV are lacking, despite promising evidence that microstate alterations occur in peripheral vertigo conditions (e.g., changes in Microstate D dynamics) [18].

Clinically, objective stratification of CV severity could support monitoring and triage in acute stroke settings. Scientifically, CV provides a model of disrupted vestibular–multisensory network coordination. We therefore tested the hypotheses that (i) severity-related microstate alterations would be most pronounced in the delta band and (ii) transitions involving MsB/MsC/MsD would differentiate moderate from severe CV. Using narrowband microstate analysis across delta–beta bands, we observed severity-dependent alterations, with delta-band transition probabilities showing the strongest associations with clinical scales and the highest discriminative performance in this cohort.

## 2. Materials and Methods

### 2.1. Participants

Fifty patients with stroke-associated vertigo, clinically defined as CV, were consecutively recruited from the Department of Neurology, Tianjin Huanhu Hospital. All CV patients were diagnosed with acute ischemic stroke involving the posterior circulation based on CT imaging, including cerebellar infarction and brainstem infarction. No cortical or hemorrhagic strokes were included. EEG recordings were obtained within one week after stroke onset. Exclusion criteria were a history of peripheral vestibular disorders, comorbid conditions affecting independent daily functioning (such as severe visual or motor impairment), and cognitive impairment or inability to cooperate with clinical assessments. Based on Dizziness Handicap Inventory (DHI) scores, CV patients were stratified into an MD group (DHI 20–60, *n* = 31) and an SV group (DHI > 60, *n* = 19). The sample size of 50 CV patients was determined based on feasibility rather than a formal power analysis, given the exploratory nature of the study. In addition, 31 age-matched healthy controls (HC) were recruited for comparison. Future studies should consider conducting a power analysis based on effect sizes observed in this study. Demographic characteristics were comparable among the MD, SV, and HC groups. No significant differences were observed in age across the three groups (Kruskal–Wallis test: *H* = 3.490, *p* = 0.175), and sex distribution was also similar among groups (*χ*^2^ = 3.347, *p* = 0.188). This study was approved by the Ethics Committee of Tianjin University (Approval No. TJU-2024-483) and the Ethics Committee of Tianjin Huanhu Hospital (Approval No. 202503241532000484034). Written informed consent was obtained from all participants prior to enrollment.

### 2.2. EEG Data Collection and Preprocessing

EEG data were acquired using a 64-channel Net Amps 400 system manufactured by Electrical Geodesics, Inc (EGI), Eugene, OR, USA [19]. Due to poor signal quality at peripheral sites, 52 electrodes were retained for analysis. Data were sampled at 1000 Hz, with electrode impedances maintained below 50 kΩ. Participants remained seated and relaxed during three resting-state blocks (20 s recording followed by 5 s rest), minimizing movement and speech. Preprocessing was performed in EEGLAB (MATLAB R2024a). Signals were band-pass-filtered between 1 and 40 Hz and downsampled to 256 Hz. Bad channels were identified and interpolated using spherical splines. For each trial, we automatically selected the first continuous 10 s segment that met a predefined quality criterion (no samples exceeding ±100 μV across retained channels); if no such segment was available, the trial was excluded. ICA (extended Infomax) was then applied to remove ocular, cardiac, and muscle artifacts.

### 2.3. Microstate Analysis

Microstate analysis was performed in MATLAB R2024a using the EEGLAB plugin MICROSTATELAB (v2.1). To investigate frequency-specific microstate dynamics, narrow-band microstate analyses were performed following broadband preprocessing. Specifically, after artifact removal and re-referencing, the continuous EEG data were band-pass-filtered into four canonical frequency bands prior to microstate segmentation: delta (1–4 Hz), theta (4–8 Hz), alpha (8–13 Hz), and beta (13–30 Hz). Band-pass filtering was implemented using zero-phase finite impulse response (FIR) filters to avoid phase distortion. For each frequency band, microstate analysis was conducted independently. Templates were derived from pooled data across all participants (HC, MD, and SV), and the same set of templates was used for all groups to avoid exaggerating group differences. Global Field Power (GFP) was computed for each participant, and EEG topographies at GFP peaks were extracted for clustering, as scalp maps are most stable and exhibit a high signal-to-noise ratio at these time points [20]. A minimum peak distance of 10 sampling points was applied, and 1000 GFP peak maps were randomly selected per participant. The selected topographies were submitted to a modified K-means clustering algorithm (modKmeans) to identify representative microstate maps. The number of clusters was tested using values from 2 to 10, and an optimal solution of four microstates was determined based on global explained variance and cross-validation criteria. Individual microstate maps were then spatially matched to the four canonical microstate templates (A–D), ignoring polarity, to ensure consistent labeling across participants. Microstate templates were back-fitted to each participant’s continuous EEG data by assigning, at each time point, the template with the highest spatial correlation. Temporal smoothing was applied to improve segmentation stability, with a minimum microstate duration of 30 ms enforced to exclude transient states. Microstate metrics were computed following standard conventions. Mean duration (ms) was defined as the average temporal length of consecutive samples assigned to a given microstate. Coverage (%) was defined as the proportion of total recording time occupied by that microstate. Mean occurrence (occurrences/s) was defined as the number of times a microstate appeared divided by the total recording duration, yielding a rate normalized per second. Global explained variance (GEV) quantified the proportion of the total EEG variance explained by each microstate class. Microstate transition probabilities were defined as the conditional probability of transitioning from one microstate to another between consecutive time points. Microstate features are reported using a structured naming scheme. Frequency bands are denoted by the prefixes Delta, Theta, Alpha, and Beta. Microstate classes are labeled MsA–MsD. Transition probability features are written as [Band]TP[From]-[To] (e.g., Delta_TP_C-B indicates the transition probability from MsC to MsB in the delta band). State-wise temporal metrics are written as [Band][Metric][State] (e.g., Delta_MO_D indicates the mean occurrence of MsD in the delta band).

### 2.4. Clinical Correlation Analysis

Spearman rank correlation was used to examine associations between clinical symptom severity and EEG microstate parameters in patients with MD and SV. Clinical measures included the Visual Analog Scale (VAS) and the Activities-specific Balance Confidence (ABC) scale. Correlations were computed between clinical scores and microstate features across delta, theta, alpha, and beta bands, including global explained variance, mean duration, time coverage, mean occurrence, and microstate transition probabilities. Only MD and SV patients were included in the analysis. Given the large number of correlation tests, *p* values were adjusted for multiple comparisons using the Benjamini–Hochberg false discovery rate (FDR) procedure (*q* = 0.05), applied separately for VAS and ABC across all tested microstate features.

### 2.5. ROC Curve Analysis

To identify the most discriminative microstate parameters as potential biomarkers for distinguishing between MD and SV, we conducted receiver operating characteristic (ROC) curve analyses on all extracted features. To ensure the robustness and generalizability of the classification performance, a stratified five-fold cross-validation procedure was applied to each parameter, and AUC values were averaged across folds. For each parameter, the area under the ROC curve (AUC) was computed, accompanied by 95% confidence intervals estimated from the distribution of fold-wise AUCs (2.5th–97.5th percentiles) under five-fold cross-validation [21]. The AUC metric provides a threshold-independent measure of classification performance, quantifying the probability that a randomly selected MD case will exhibit a higher parameter value than a randomly selected SV case (or vice versa, depending on the direction of effect).

### 2.6. Statistical Analysis

Statistical analyses were performed using MATLAB R2024a and GraphPad Prism 9.5.1 (GraphPad Software). Normality of continuous variables was assessed using the Shapiro–Wilk test [22]. Given the mixed distributional properties of microstate parameters, parametric analyses with variance correction were applied when appropriate. Group differences in EEG microstate parameters were examined using two-way repeated-measures ANOVAs [23], with group (HC, MD, SV) as the between-subject factor and microstate class (A–D) as the within-subject factor. Analyses were conducted separately for each frequency band and included temporal microstate parameters and transition probabilities. Significant effects were followed by Holm-Šídák’s post hoc tests. For pairwise comparisons between MD and SV, independent-samples *t*-tests with Welch’s correction were applied when variance homogeneity was violated, with Mann–Whitney U tests used as robustness checks. Clinical correlations were assessed using Spearman rank correlation. All statistical tests were two-tailed, and significance thresholds were defined as follows: ns (not significant, *p* ≥ 0.05), * *p* < 0.05, ** *p* < 0.01, *** *p* < 0.001, and **** *p* < 0.0001.

## 3. Results

### 3.1. Microstate Topographies and Dynamic Parameters

**Figure 1:**
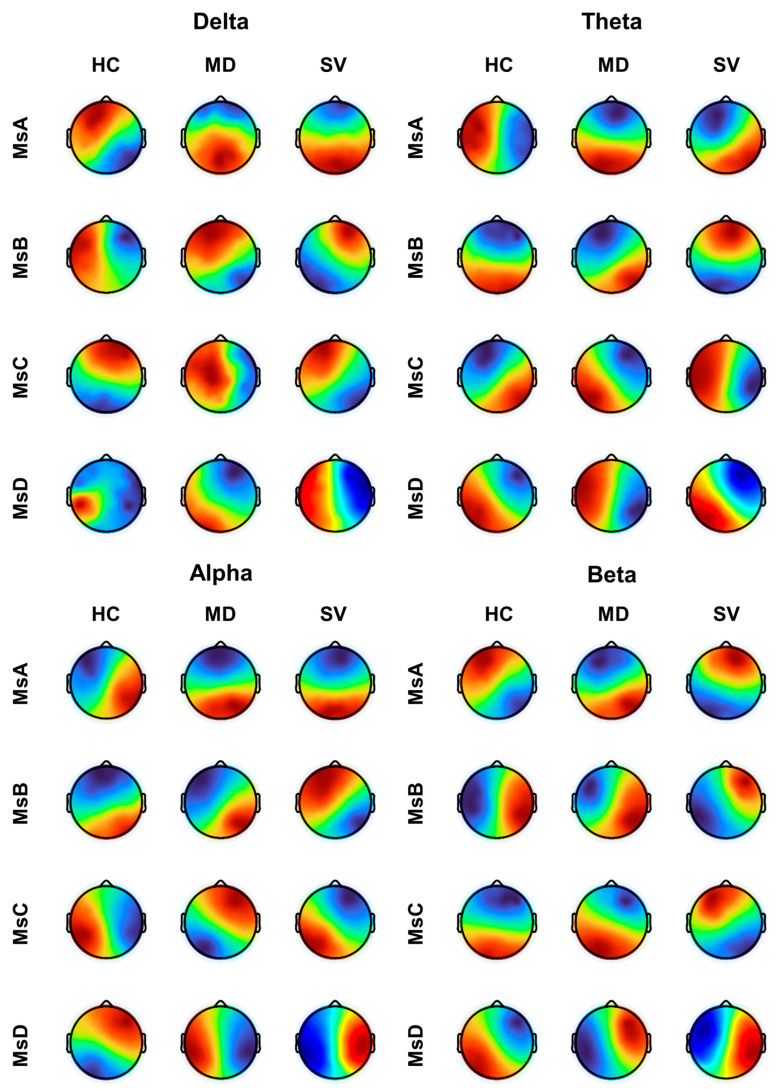
Frequency-specific microstate topographies across groups. Representative microstate scalp topographies (MsA to MsD) derived from narrowband EEG in the delta, theta, alpha, and beta bands. The maps represent group-level consensus microstate templates obtained from all participants (HC, *n* = 31; MD, *n* = 31; SV, *n* = 19) using k-means clustering. Color scale: Red indicates positive potential and blue indicates negative potential.

Across frequency bands, microstate centroid topographies showed a severity-related reconfiguration from HC to MD to SV (Figure 1). In HC, most classes exhibited comparatively stable left–right dipolar patterns with relatively focal extrema. In MD, several classes—most consistently MsA and MsD—shifted toward more pronounced anterior–posterior gradients, indicating a redistribution of the dominant field from hemispheric lateralization to fronto-occipital opposition. SV demonstrated the most marked morphological alteration, characterized by stronger hemispheric segregation and frequent polarity/lateralization reorganization (most evident in MsB–MsD), with sharper transitions between opposite fields. Band-specific inspection suggested that delta/theta changes were driven mainly by MsA (with additional displacement in MsB/MsD), whereas alpha and beta displayed the clearest group separation, showing broader extrema shifts and mirror-like hemispheric redistribution in SV.

**Figure 2:**
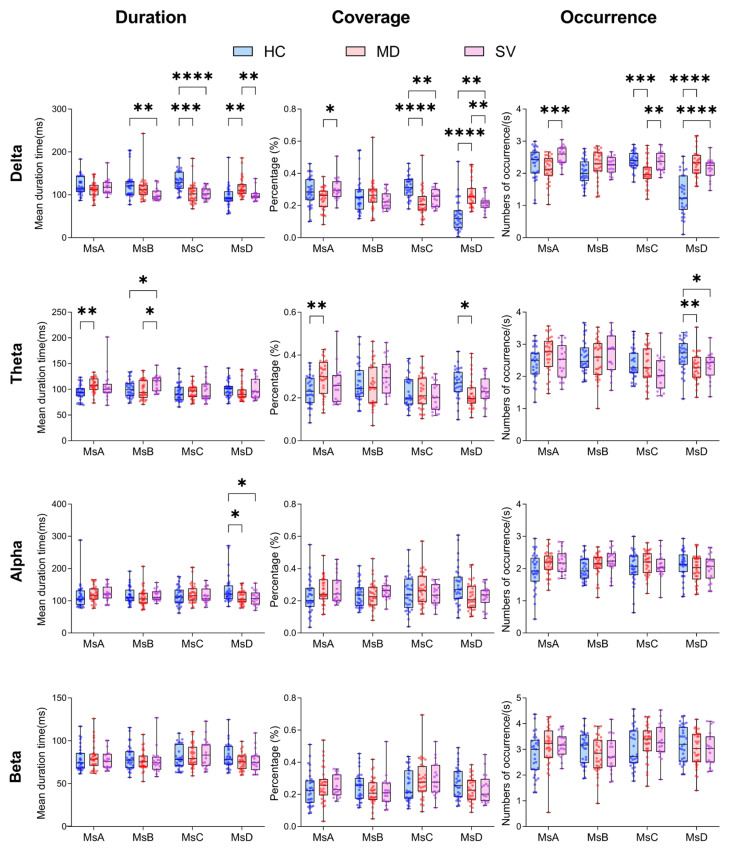
Group differences in microstate parameters across narrowband frequencies. Box plots show group comparisons of microstate mean duration, coverage, and mean occurrence for each narrowband frequency band (delta, theta, alpha, and beta) and each microstate class (MsA to MsD) (HC, *n* = 31; MD, *n* = 31; SV, *n* = 19). Boxes indicate the interquartile range with the median, and points represent individual participants. Group differences were assessed separately for each frequency band using two-way repeated-measures ANOVA with group (HC/MD/SV) as the between-subject factor and microstate class (A–D) as the within-subject factor, followed by Holm–Sidak post hoc tests when applicable.

Two-way repeated-measures ANOVA revealed significant group × microstate interactions across frequency bands, with the most robust effects observed in delta and theta bands (Figure 2). In the delta band, HC participants demonstrated a distinctive pattern characterized by enhanced MsC activity and suppressed MsD activity relative to clinical groups. Specifically, HC showed significantly longer MsC mean duration (vs MD: +26.53 ms, *p* = 0.0001; vs. SV: +25.99 ms, *p* < 0.0001), higher coverage (vs MD: +0.097, *p* < 0.0001), and increased mean occurrence (vs MD: +0.373, *p* = 0.0002), while exhibiting shorter MsD mean duration (vs MD: −22.92 ms, *p* = 0.0012), reduced coverage (vs MD: −0.137, *p* < 0.0001), and decreased mean occurrence (vs MD: −0.963, *p* < 0.0001; vs. SV: −0.825, *p* < 0.0001). Corresponding alterations in transition probabilities emerged, with MD displaying reduced A-C transitions (−0.182, *p* < 0.0001) and elevated A-D transitions (+0.174, *p* < 0.0001) compared to HC. The theta band revealed a systematic reorganization of microstate dynamics in clinical populations, manifested through altered MsA parameters (HC vs. MD: shorter mean duration −12.80 ms, *p* = 0.003; lower coverage −0.061, *p* = 0.006) and comprehensive transition probability shifts. HC participants exhibited reduced A-B transitions (vs MD: −0.224, *p* < 0.0001; vs. SV: −0.287, *p* < 0.0001), elevated A-D transitions (vs both groups: +0.263, *p* < 0.0001), enhanced B-C transitions (vs MD: +0.216, *p* < 0.0001; vs. SV: +0.235, *p* < 0.0001), and diminished C-D transitions (vs both groups: approximately −0.158, *p* < 0.0001). Alpha-band effects were circumscribed to MsD mean duration prolongation in non-MD groups (HC and SV vs. MD: +23.03 and +24.62 ms, *p* = 0.036) and selective transition probability modifications. These convergent findings identify delta-band MsC/MsD parameters and theta-band transition dynamics as candidate neurophysiological biomarkers for CV, potentially reflecting fundamental disruptions in low-frequency neural oscillations underlying default mode network function.

### 3.2. Correlation Between Microstate and Clinical Characteristics

**Figure 3:**
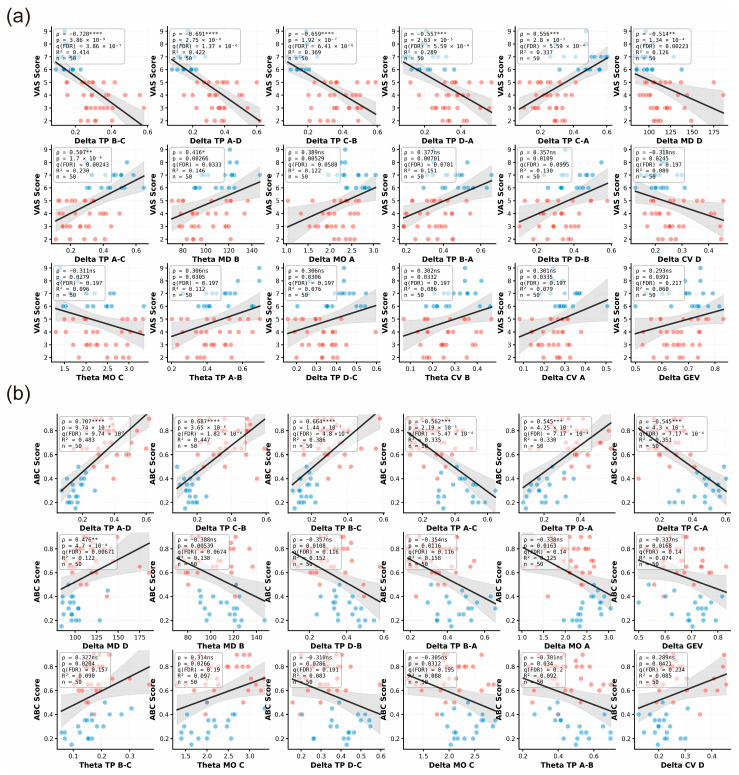
Associations between frequency-specific EEG microstate metrics and clinical scores. (**a**) Scatterplots showing the relationships between the VAS score and selected narrowband microstate features. (**b**) Scatterplots showing the relationships between the ABC score and selected narrowband microstate features. Only MD and SV patients were included (MD, red, *n* = 31; SV, blue, *n* = 19; total *n* = 50). Associations were tested using Spearman’s rank correlation (two-tailed). *p*-values were additionally adjusted using Benjamini–Hochberg FDR correction; *q* (FDR) values are shown in the insets, and *q* < 0.05 was considered significant. Insets report Spearman’s rank correlation coefficient (ρ) with two-tailed *p* value and the coefficient of determination (R^2^) for the linear fit.

To assess the relationship between EEG microstate dynamics and clinical manifestations of cervicogenic vertigo, we examined correlations between clinical scores (VAS and ABC) and frequency-specific microstate features in MD and SV patients using Spearman correlation with false discovery rate (FDR) correction. After FDR adjustment (*q* < 0.05), both clinical scales showed robust associations predominantly involving delta-band microstate transition probabilities (Figure 3).

Higher VAS scores were most strongly associated with reduced delta-band transitions from microstate B to C (*ρ* = −0.720, *q* = 3.86 × 10^−7^), A to D (*ρ* = −0.691, *q* = 1.37 × 10^−6^), and C to B (*ρ* = −0.659, *q* = 6.41 × 10^−6^), whereas a positive association was observed for transitions from C to A (*ρ* = 0.556, *q* = 5.59 × 10^−4^, Figure 3a). Similarly, ABC scores were positively correlated with delta-band transitions from A to D (*ρ* = 0.707, *q* = 9.74 × 10^−7^) and from C to B (*ρ* = 0.687, *q* = 1.82 × 10^−6^, Figure 3b), indicating that altered delta-band microstate transition dynamics are closely linked to both symptom severity and balance impairment.

### 3.3. ROC Curve Analysis for Optimal Discriminative Feature Determination

**Figure 4:**
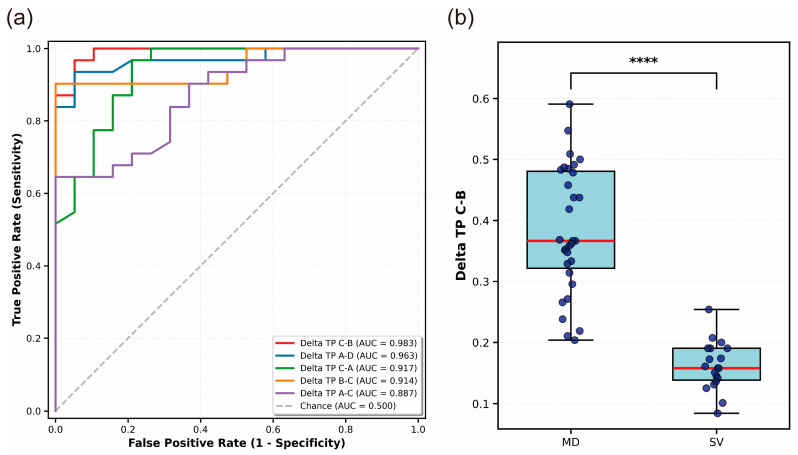
ROC curve analysis and group comparison for MD vs. SV classification. (**a**) ROC curves for the top five delta band microstate transition probability features (MD, *n* = 31; SV, *n* = 19). The diagonal dashed line represents chance-level performance. AUCs are shown with 95% confidence intervals estimated from five-fold cross-validation (2.5th–97.5th percentiles of fold-wise AUCs). All features exhibited excellent classification ability. (**b**) Distribution of Delta_TP_C-B values in MD and SV groups (MD, *n* = 31; SV, *n* = 19). MD patients showed significantly higher values than SV patients. Group differences were tested using Welch’s t-test, with Mann–Whitney U test used as a nonparametric confirmation.

To identify neurophysiological markers that could differentiate MD from SV, we performed comprehensive ROC curve analysis on EEG microstate parameters spanning four frequency bands. Remarkably, the top five discriminative features all originated from delta-band microstate transition probabilities (Figure 4a), demonstrating the particular relevance of slow-wave brain dynamics in severity classification of central vertigo. The transition probability from MsC to MsB (Delta_TP_C-B) emerged as the most powerful discriminator, achieving classification performance with an AUC of 0.983 (95% CI: 0.925–1.000, *p* = 8.42 × 10^−6^). This was followed by Delta_TP_A-D (AUC = 0.963, 95% CI: 0.881–1.000, *p* = 5.02 × 10^−5^), Delta_TP_C-A (AUC = 0.917, 95% CI: 0.692–1.000, *p* = 2.97 × 10^−3^), Delta_TP_B-C (AUC = 0.914, 95% CI: 0.690–1.000, *p* = 3.04 × 10^−3^), and Delta_TP_A-C (AUC = 0.888, 95% CI: 0.740–0.996, *p* = 1.30 × 10^−3^). All five delta-band transition features demonstrated excellent and stable classification performance under five-fold cross-validation (AUC > 0.85), substantially outperforming chance-level prediction (AUC = 0.500, represented by the diagonal reference line in Figure 4a).

Given its superior discriminative performance, we conducted detailed characterization of the Delta_TP_C-B feature (Figure 4b). Independent-samples t-test revealed that MD patients (*n* = 31) displayed significantly higher Delta_TP_C-B values (Mean = 0.383, SD = 0.104) compared to SV patients (*n* = 19; Mean = 0.162, SD = 0.040), yielding a highly significant difference (*t* = 8.89, *p* = 1.04 × 10^−11^). This finding was corroborated by non-parametric Mann–Whitney U test (*U* = 584.0, *p* = 7.59 × 10^−9^), confirming the robustness of the effect across distributional assumptions. The magnitude of this difference was large (Cohen’s d = 2.59), indicating minimal overlap between the two clinical populations. The present effect size of 2.59 thus reflects a substantial neurophysiological distinction between MD and SV patient groups, consistent with the excellent AUC value.

## 4. Discussion

The present study demonstrates that EEG microstate analysis—particularly within the delta frequency band—provides powerful discriminative capability for assessing the severity of CV. The key finding that the delta-band transition probability from MsC to MsB achieved excellent classification performance (AUC = 0.983) between moderate and severe vertigo highlights the potential of dynamic EEG markers as objective biomarkers for central vestibular dysfunction. This result addresses a critical clinical gap in a condition traditionally resistant to reliable quantification. Microstates have long been conceptualized as the fundamental “atoms of thought,” reflecting momentary global configurations of large-scale brain networks [24,25], and subsequent methodological advances have established their robustness and interpretability in resting-state EEG [16].

The prominence of the Delta_TP_C-B invites mechanistic interpretation. MsC has been consistently linked to the salience network and anterior components of the default mode network, supporting interoceptive processing and internal state monitoring, whereas MsB is associated with visual and posterior parietal networks involved in visuospatial attention and sensory-guided orientation [25,26,27]. Transitions between these states likely reflect the dynamic reweighting between internally generated vestibular signals and external visual reference frames—a process that is central to spatial orientation and postural control. The functional relevance of such transitions is supported by evidence that microstate sequences encode structured, non-random information flow across cortical networks [28,29].

The selective involvement of delta-band dynamics is consistent with the role of slow oscillations in large-scale network coordination and long-range communication. Delta activity has been repeatedly implicated in the integration of distributed cortical systems and top-down modulation during both rest and pathological states. Pathologically increased waking delta activity is a well-established marker of cortical dysfunction and disrupted thalamocortical interactions, as demonstrated across neuropsychiatric and neurodegenerative disorders [16,28,30]. In CV, where pathology may involve vestibular brainstem nuclei, cerebellar circuits, or thalamocortical projections to the parieto-insular vestibular cortex, altered delta-band coordination is therefore expected [4].

Importantly, the superior performance of transition probabilities over static spectral measures suggests that disease severity is better captured by impaired temporal coordination between networks rather than by changes in regional activation alone. This observation aligns with accumulating evidence that microstate dynamics, including mean duration, mean occurrence, and transition structure, are more sensitive indicators of network dysfunction than conventional power-based metrics [28]. The C–B transition may specifically index the efficiency of reorienting from interoceptive vestibular processing toward visuospatial stabilization. As central vestibular impairment worsens, this reorientation process appears increasingly dysregulated, a disruption robustly captured by delta-band microstate dynamics.

Several mechanisms may underlie the observed delta-band alterations [31]. Disruption of ascending vestibular pathways could directly perturb thalamocortical oscillatory dynamics, particularly within relay nuclei projecting to multisensory cortical regions involved in vestibular integration. Additionally, compensatory cortical reorganization following vestibular dysfunction may alter interactions between visual, salience, and attentional networks, manifesting as abnormal microstate transitions. Similar compensatory or maladaptive network reconfigurations have been documented in aging, mild cognitive impairment, and dementia using microstate analysis [31,32,33]. Finally, the diffuse cognitive and affective consequences of chronic vertigo may further contribute to altered slow oscillatory coordination across distributed networks, consistent with the broader clinical relevance of transient brain states for diagnosis and prognosis [34].

The high classification accuracy achieved using a single resting-state EEG parameter has important clinical implications. CV currently lacks objective severity markers, relying largely on subjective symptom reports and bedside examinations that may be insensitive to cortical dysfunction. Resting-state EEG is rapid, inexpensive, and requires minimal patient cooperation, making it well suited for acute or severely symptomatic patients. Microstate-based biomarkers have already shown promise in schizophrenia, neurodegenerative disease, and altered states of consciousness [35,36,37], and the present findings extend this framework to vestibular disorders.

Several limitations should be acknowledged. First, the present findings require replication in larger, independent cohorts to establish generalizability. Second, the cross-sectional design precludes causal inference or prognostic conclusions; longitudinal studies are needed to determine whether microstate dynamics evolve with recovery or predict long-term clinical outcomes. Finally, although all patients had posterior circulation ischemic stroke, lesion locations were heterogeneous, involving either the cerebellum or brainstem. This anatomical variability may differentially affect large-scale cortical dynamics and contribute to inter-individual variability in microstate measures, which could not be disentangled in the current sample. Future studies integrating lesion-symptom mapping and multimodal neuroimaging approaches may help clarify the anatomical and network-level mechanisms underlying microstate alterations in central vertigo [34,38].

## 5. Conclusions

This investigation highlights the potential of EEG microstate analysis for characterizing central vestibular dysfunction and suggests that delta-band transition probabilities—particularly the C-B transition—may provide useful discriminative capacity for severity stratification. While the classification accuracy achieved by this single parameter shows promise, further validation is needed to confirm its effectiveness as an objective biomarker for a condition that has historically defied quantification. The involvement of delta-band dynamics and C-B transitions implies that CV severity may reflect disrupted coordination between salience-interoceptive and visuospatial processing networks, which aligns with the role of vestibular function in integrating internal and external spatial references. These findings open potential avenues for more objective vestibular assessment, prognostication, and therapeutic monitoring, while providing insight into the cortical network dynamics underlying vestibular-multisensory integration.

## Data Availability

The data presented in this study are available upon request from the corresponding author due to privacy and ethical restrictions involving human participant data.

## Abbreviations

The following abbreviations are used in this manuscript:

ABC	Activities-specific Balance Confidence (scale)
ANOVA	Analysis of variance
AUC	Area under the curve
CV	Central vertigo
DHI	Dizziness Handicap Inventory (score)
EEG	Electroencephalography
GFP	Global Field Power
GEV	Global explained variance
HC	Healthy control
MD	Moderate vertigo (group)
MsA–MsD	Microstate class A–D
ROC	Receiver operating characteristic
SD	Standard deviation
SV	Severe vertigo (group)
VAS	Visual Analog Scale
